# Molecular Dynamics Simulations of the Spike Protein Receptor Binding Domain Adsorption to Material Surfaces

**DOI:** 10.1021/acs.jpcb.5c04391

**Published:** 2025-10-22

**Authors:** Mohammed A. Haider Farouq, Karina Kubiak-Ossowska, Mohammed M. Al-Qaraghuli, Valerie A. Ferro, Paul A. Mulheran

**Affiliations:** † Department of Chemical and Process Engineering, 3527 University of Strathclyde, 75 Montrose Street, Glasgow G1 1XJ, U.K.; ‡ Department of Physics/Archie-West HPC, 3527 University of Strathclyde, 107 Rottenrow East, Glasgow G4 0NG, U.K.; ∥ Strathclyde Institute of Pharmacy and Biomedical Sciences, University of Strathclyde, 161 Cathedral Street, Glasgow G4 0RE, U.K.

## Abstract

The receptor binding domain (RBD) of the SARS-CoV-2 spike protein is an important diagnostic and therapeutic target since it binds to the peptidase domain of the angiotensin-converting enzyme 2 (ACE2) receptor, thus facilitating infection by the virus. Many diagnostics utilize the adsorption of proteins onto material surfaces and nanoparticles to create functional couples. In this work, the adsorption of the histidine tag (His-Tag) modified RBD on various inorganic surface models is explored by using fully atomistic molecular dynamics simulations. The material surfaces used are an experimentally relevant negatively charged silica surface, a model positively charged surface, and a self-assembled monolayer terminated with negatively charged carboxyl groups. The simulations with both negatively charged surface models show the protein adsorbing rapidly and specifically, while the protein does not adsorb on the positively charged surface model. Adsorption of the His-Tag modified RBD on both negative surfaces is also favorable for device manufacture, with the protein retaining its structure while its ACE2-binding residues remain free to interact with the environment due to its orientation in the adsorbed state. Consequently, these results can guide the development of new diagnostics through the choice of substrate and protein modification.

## Graphical abstract

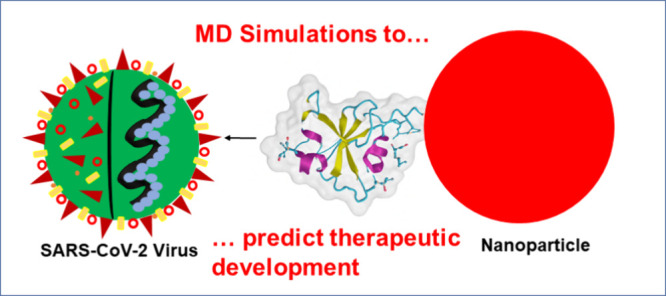

## 1 Introduction

Studying protein adsorption onto material surfaces can indicate the suitability of the material for use in a therapeutic or diagnostic application.^1^ The spike (S) protein is one of four main structural proteins in the SARS-CoV-2 virus, along with the envelope (E), membrane (M), and nucleocapsid (N) proteins (Figure 1). The M and E proteins are involved in virus morphogenesis and assembly,^2^ while the N protein protects the RNA at the virus’s core. The S protein is on the outside and is the entry point of the virus into host cells.

**Figure 1:**
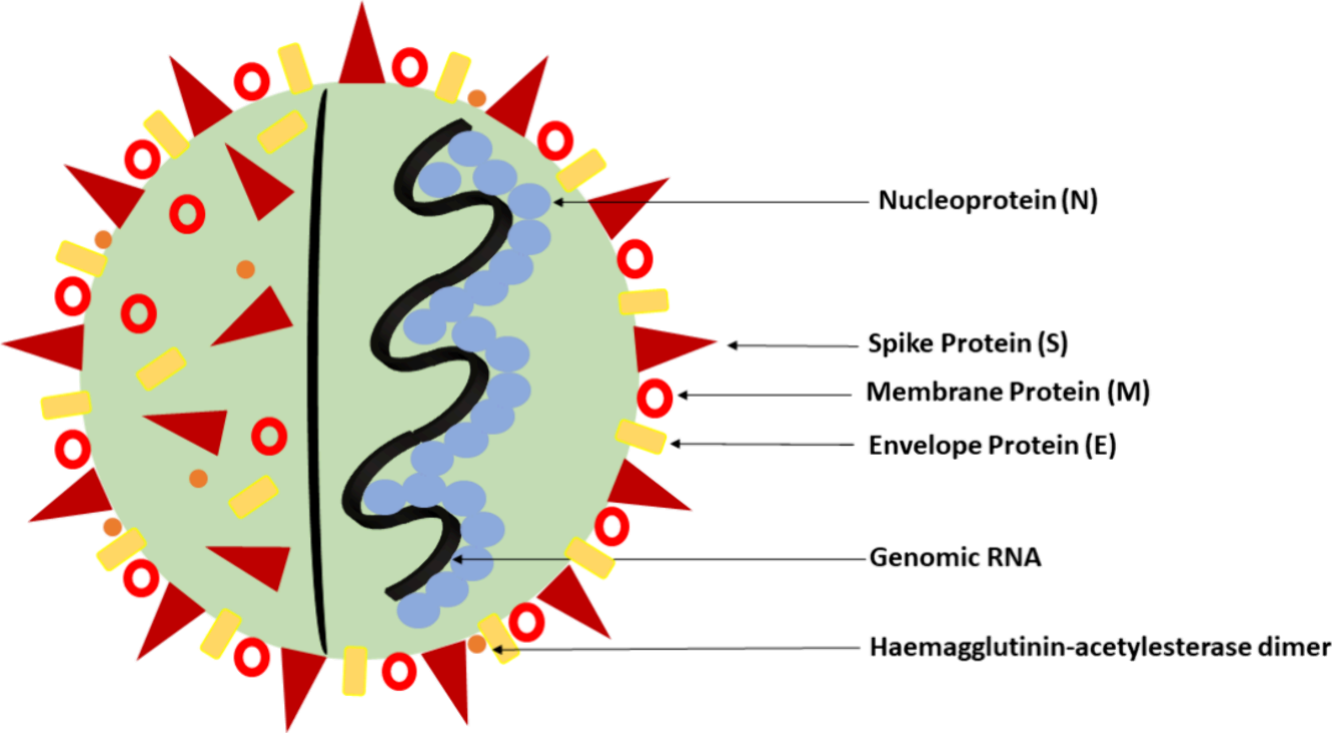
Diagram illustrating the structural proteins of the SARS-CoV-2 virus.

The S1 subunit of the S protein contains the receptor-binding domain (RBD, Figure 2a) that binds to the angiotensin-converting enzyme 2 (ACE2) receptor and initiates infection by the virus; therefore, the S protein has been used in several therapeutic applications against the SARS-CoV-2 virus. For example, the immediate response to the COVID-19 pandemic saw an mRNA-based vaccine, targeting the S protein, developed by BioNTech in collaboration with Pfizer.^3^ Another mRNA-based vaccine targeting the S protein was also developed at speed by Moderna.^4^ The viral vector Oxford-AstraZeneca COVID-19 vaccine^5^ used a modified, replication-deficient chimpanzee adenovirus as a vector. Essential replication genes were deleted from the adenovirus and replaced with a gene encoding the S protein.^6,7^

**Figure 2:**
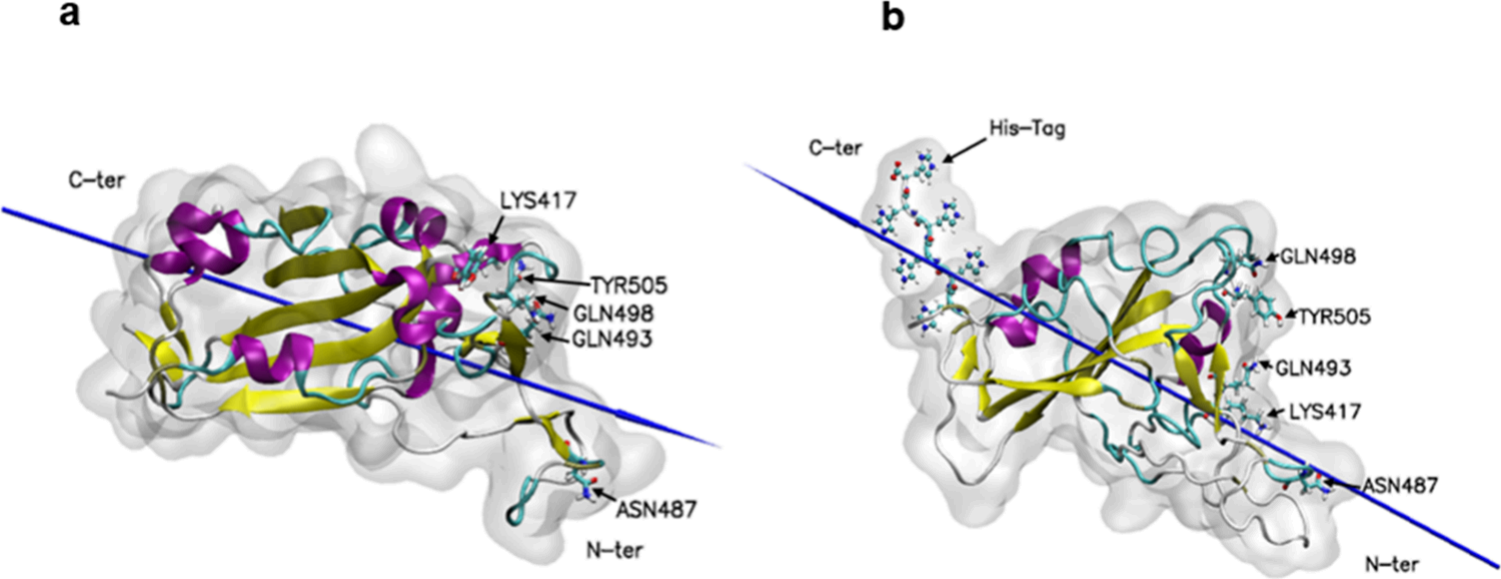
(a) RBD of the S protein, taken from PDB entry 7BWJ and illustrated using VMD 1.9.4.^8^ The protein is indicated as a ghost surface, while the secondary structural α-helices are colored purple and the beta sheets are colored yellow. (b) RBD modified with a six-residue His-Tag. The His-Tag is added to the C-terminus of the protein and is shown in a CPK representation. Lys417, Asn487, Gln493, Gln498 and Tyr505 are indicated by the ‘liquorice’ representation and colored by atom type (carbon is cyan, nitrogen is blue, oxygen is red, and hydrogen is white). The blue needle indicates the dipole moment of the proteins.

Molecular dynamics (MD) simulations can provide detailed insight at an atomistic level into the complex structure of proteins and have previously provided information regarding interactions of the S protein with different model surfaces. For instance, Malaspina and Faraudo^9^ investigated the full S protein interactions with model cellulose and graphite surfaces, successfully establishing the strength of binding by protein–surface contacts and hydrogen bonds.^9^ Brodowski et al.^10^ also used MD simulations to model the ACE2 receptor on a functionalized sensor, and analyzed the conformations by stability and statistical data. The effect of adsorption of the ACE2 receptor on silane monolayers was also studied.^11^ The ACE2 receptor was found to maintain its bioreactivity while displaying increased interactions with the S protein RBD on adsorption to the monolayers with silane molecules, short alkyl chains, positively charged head-groups, and silane molecules without charged head-groups.^11^ Lecot et al.^12^ also studied the adsorption of a streptavidin–biotin complex on silica surfaces with five different self-assembled monolayers (SAMs). The alkyl chain length and the headgroup charge of the silane molecules directly influenced conformational change and mobility of the streptavidin.

The analyses gathered from MD simulations can guide specific and site-directed optimization of protein adsorption onto nanomaterials, allowing engineering of new biotherapeutics. A His-Tag is a string of histidine residues fused to a recombinant protein to help with purification.^13^ The ability of a His-Tag to bind several types of nanoparticles and metal ions means not only can the protein be easily purified and detected, but the adsorption can also be exploited for the development of new biotechnology.^14^ The availability of the crystal structure of the S1 subunit has provided a route to optimize the conjugation of the protein domains with nanoparticles, and the addition of a His-Tag to the N or C-terminal of the protein may enhance the protein’s ability to conjugate to negatively charged nanoparticles.

In this work, the RDB protein (Figure 2a) was modified with a His-Tag (Figure 2b). In Figure 2, the dipole moment of the proteins is displayed, since it is a strong indicator of how the protein will adsorb on charged material surfaces as first proposed by Jiang and co-workers.^15,16^ Here the His-Tag protein interactions are simulated with three inorganic surfaces: (i) a negatively charged siloxide-rich silica surface; (ii) a positively charged model Si-rich surface; and (iii) a negatively charged surface created by SAMs. These models were used due to their experimental relevance and to establish the role that surface charge plays in orienting the adsorbed protein.

SAMs are ordered formations of molecules formed by the adsorption of an active surfactant to a solid surface. These molecules bind to the surface in an ordered way and can have various head-groups that control the physicochemical properties of the interface they present to the solution.^17^ SAMs have been widely used in the past to study the adsorption of proteins both experimentally^18–20^ and computationally,^21,22^ proving to be effective surfaces for functionalization with proteins, enabling a variety of applications.

The RBD has been found to bind to the ACE2 receptor through various residues, including Lys417, Asn487, Gln493, Gln498, and Tyr505.^23^ Therefore, in this work, we monitor the positioning of these residues in the adsorbed protein, with the view that these residues must be free to interact with the solution if the protein is to retain its ability to bind to the ACE2. Furthermore, the adsorbed protein must maintain its native conformation upon adsorption to retain its functionality. We will show that negatively charged surfaces at pH6 promote such favorable protein adsorption, thus guiding the development of new therapeutics and diagnostics through suitable material selection and processing conditions.

## 2 Computational Details

### 2.1 MD Simulations

The crystal structure of the RBD (PDB: 7BWJ)^24^ of the S protein was modified with a six-residue His-Tag at the C terminus, and particular residues were protonated using the ProteinPrepare^25^ web application within the PlayMolecule Viewer plugin (open.playmolecule.org), to run the simulations at pH6 (see Table 1). The simulations were performed at pH 6 to ensure the His-Tag is protonated and is able to enhance the protein’s ability to conjugate to negatively charged nanoparticles, making it more likely to result in an optimum orientation of the conjugated protein for use in new therapeutics. The final modified structure contained 200 amino acids (1638 atoms, residues 333–532), and a net protein charge at pH 6 of +11e due to its distribution of charge residues (including those listed in Table 1).

**Table 1:** List of Residues That Were Protonated To Run the His-Tag Modified RBD Simulations at pH 6

**residue type**	**residue number**
Asp	398, 406
His	519, 527, 529, 530, 531, 532

The NAMD 3.0 package^26^ was utilized along with the Charmm-27 force field, and the simulation results were analyzed with VMD software.^8^ The simulations were performed in three stages following a previously reported protocol.^27^ The first stage involved water (TIP3P model)^28,29^ and ions being added to the simulation cell, which already contained the static, tagged protein obtained from the PDB structure. This was followed by water and ion minimization of 1000 steps and a subsequent 100 ps run in a constant pressure/temperature (NPT) ensemble, with an integration time-step of 1 fs, at a target temperature of 300 K and a pressure of 1 atm. The second stage simulated the complete system consisting of protein, water, and ions through energy minimization for 10,000 steps, followed by NPT equilibration for 300 ps. The final stage consisted of an initial 10 ns run, with a 2 fs time-step at 300 K in the constant volume/temperature (NVT) ensemble, extended to give a 100 ns production trajectory. The Langevin thermostat, Langevin piston Nose-Hoover method barostat, and periodic boundary conditions (PBC) were used along with the SHAKE algorithm, and the cutoff distance for the van der Waals interactions was set at 12 Å. Particle mesh ewald (PME) summation was used to describe the electrostatic Coulomb interactions.^30^ The setups differed slightly between simulations; additional details for each system are given below.

#### 2.1.1 Simulation in Bulk Water

The protein was solvated in a rectangular water box extending at least 15 Å from the protein surface (40,379 water molecules) and neutralized by adding 7 Na^+^ and 18 Cl^–^ ions (yielding 0.02 mol/L ionic strength solvent), resulting in a system with 58,887 atoms. The simulation of the protein in water was performed for 100 ns to provide a reference trajectory and protein structure.

#### 2.1.2 Simulation with SiO_2_ Surfaces

The SiO_2_ slab, created from an α-cristobalite structure, was modeled as ions fixed in space.^31^ An electric field is induced through the simulation box,^32^ so that the electrostatic environment above the slab mimics that expected above a negatively charged silica surface. The slab dimension was 103 Å × 199 Å × 13 Å, with 17,280 atoms, yielding two different faces: (i) a SiO_2_ surface with siloxide (SiO^–^) groups at the top of the slab and (ii) undercoordinated Si species at the bottom. The Charmm-27 force-field parameters for the surface were used according to Patwardhan and co-workers,^33^ giving a negatively charged surface with siloxide groups exposed at an areal density comparable to experimental systems,^31^ and a model positively charged surface with under-coordinated Si species exposed.

In the silica siloxide simulation, the protein was placed ∼44 Å above the surface (the protein surface separation varied between 44 and 50 Å) and solvated in a rectangular water box extending at least 40 Å beyond the protein in the *x*-axis (∼65,023 water molecules), resulting in a system with ∼214,323 atoms. The system was neutralized, and the NaCl concentration was set to 0.08 mol/L, which added 277 Na^+^ and 288 Cl^–^ ions and shielded the charged silica surfaces in the simulation.

In the silica undercoordinated system, given the net charge and the influence of the His-Tag, the protein was positioned closer to the surface, with the His-Tag facing away from the positive surface to encourage adsorption. The distance from the surface was ∼22 Å (the protein surface separation varied between 22 and 24 Å), and the system was solvated in a rectangular box extending at least 44 Å beyond the protein (∼77,137 waters) in the *x*-axis. As above, the system was neutralized and the ionic concentration was set to 0.08 mol/L. This added 277 Na^+^ and 288 Cl^–^, yielding a system with ∼250,665 atoms.

#### 2.1.3 Simulation with the SAM Surface

The SAM surface was constructed with two carboxyl-terminated layers, with the head-groups facing out to solution. All the carboxyl molecules were in their deprotonated (−COO^–^) state, a suitable model at pH6, which is much higher than the carboxyl p*K*_a_; this creates a homogeneous negative surface. The molecules consisted of a backbone of four carbon molecules in addition to the carboxyl groups, and the broken C–C bonds were patched with hydrogen atoms to satisfy the valence requirements. The individual molecules were placed parallel to each other with a distance of 4.97 Å between them,^34^ and the slab contained 15,283 atoms with dimensions of 107 Å × 147 Å × 77 Å. The thickness of each layer was larger than the 12 Å cutoff distance of the van der Waals forces, in order to prevent artificial interaction with the next layer. The first carbon atom of every SAM molecule was fixed in space, leaving the other three carbons on the backbone and the terminal functional groups free to move. The production trajectory was computed in the NPT ensemble (1.01325 bar atmospheric pressure, isotropic), with a time step of 2 fs at 300 K. The protein was positioned ∼20 Å above the surface (the protein surface separation varied between 20 and 23 Å), and solvated in a rectangular box extending at least 60 Å (∼36,824 water molecules) in the *z* axis. The system was neutralized, and NaCl concentration was set to 0.2 mol/L. This added 1113 Na^+^ and 138 Cl^–^ ions, shielding both COO^–^ surfaces and resulting in a system with ∼125,864 atoms. The higher ionic strength of 0.2 mol/L was chosen for the SAMs surface due to its influence on the system charge.

### 2.2 RMSD, RMSF, Hydrogen Bonds, and Radius of Gyration

The root-mean-square deviation (RMSD) and root-mean-square fluctuations (RMSF) analyses were conducted using custom Tcl scripts that were executed through the Tk Console of VMD.^8^ RMSD, which is widely used in bioinformatics, quantifies the structural variability of a protein relative to a reference structure.^35^

The RMSD is defined as

$$R_{D}(t)=\sqrt{\frac{\sum_{i=1}^{N_{a}}|{\vec{r}}_{i}(t)-{\vec{r}}_{i}(0){|}^{2}}{N_{a}}}$$ 1

where *N*_a_ is the number of backbone C atoms in the protein structure and ${\vec{r}}_{i}(t)$ is the position of the *i* ^th^ atom at a given time *t*. To calculate the RMSD, the two protein structures to be compared are treated as rigid bodies and overlapped using translations and rotations. Additionally, VMD^8^ also has some built-in RMSD analysis tools used to overlap protein structures and double-check the values obtained via the tcl scripts.

The RMSF is the RMSD calculated for each protein residue, and it is referred to as “fluctuations” because it reflects each residue’s mobility during the MD trajectory. The RMSF reports the residue movement from its average position over the entire length of the MD trajectory. The time average fluctuations of atoms belonging to the same residue were calculated from the formula:

$$R_{F}(i)=\sqrt{\left\langle \frac{\sum_{i=1}^{N_{k}}|{\vec{r}}_{i}(t)-\langle {\vec{r}}_{i}\rangle {|}^{2}}{N_{k}}\right\rangle }$$ 2

where ${\vec{r}}_{i}(t)$ is the position of atom *i* in residue *k* at time *t*, *N*_*k*_ is the number of atoms in residue *k*, and ⟨ · ⟩ is the time average over the trajectory. As with RMSD, an additional component to RMSF is introduced if two domains and/or chains change their relative orientations; therefore, the scripts were optimized to focus on each protein fragment to omit these effects. The most frequently used unit for RMSD and RMSF is Å (10^–10^ m), as it is convenient for the protein length scale.

Hydrogen bond formation within the protein is an important indicator for protein stability and motility in simulation.^36^ We therefore monitored the number of intramolecular hydrogen bonds involving the protein through the simulations. The radius of gyration (RoG) measures the compactness of a protein structure and is an important measure to elucidate the stability of the protein in simulation, along with the RMSD. Both hydrogen bonding and RoG data were computed through plugins built into VMD.^8^

## 3 Results and Discussion

### 3.1 Simulation in Water

The simulation in solution is important for ensuring the stability of the protein with the force field and molecular dynamics protocol employed. To establish whether the protein maintains its structural integrity in the simulation, it was placed in a periodic box with water molecules and NaCl ions as described above, and the computation of a 100 ns trajectory was performed.

The optimal overlap between the protein structure after energy minimization and that at the end of the simulation is shown in Figure 3a. It is apparent that the main secondary structure elements are unchanged and that the tertiary structure is stable. The loop regions show more flexibility, and the His-Tag has the most deviation between initial and final structure, as might be expected.

**Figure 3:**
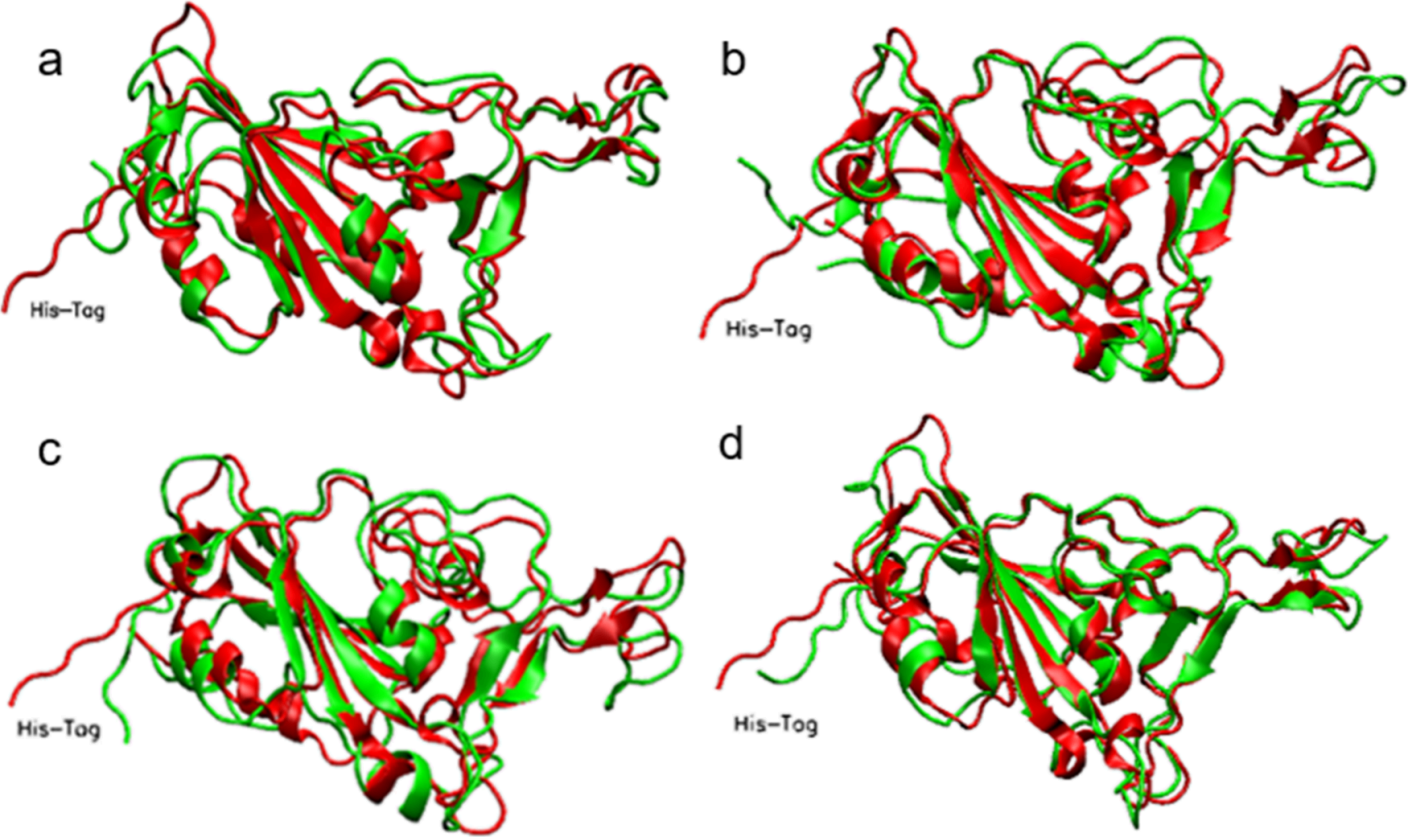
Structure overlaps of the protein after 100 ns simulation in (a) water, (b) with a siloxide-rich silica surface, (c) with a silica undercoordinated surface, and (d) with a SAM surface. The structure after energy minimization is indicated in red, while the structure after 100 ns is indicated in green, and the His-Tag is annotated.

The RMSD of the protein (Figure 4a) is initially ∼2 Å because of the protein minimization and equilibration computed prior to the production trajectory, indicating a small deviation from the reference native 3D structure, signifying that the protein is stable. As the simulation in water progresses, the situation changes ∼55 ns into the trajectory when the RMSD increases to ∼4.4 Å (Figure 4a). This shift after 55 ns, when compared to the simulation beforehand, is attributed to the bending and twisting of the protein through the loops connecting the subsequent modules, as observed in the simulation trajectory (see SI) and apparent in Figure 3a. After this small increase, the RMSD remained reasonably constant for the last 30 ns of the simulation, suggesting that the protein structure is not undergoing any substantial structural changes.

**Figure 4:**
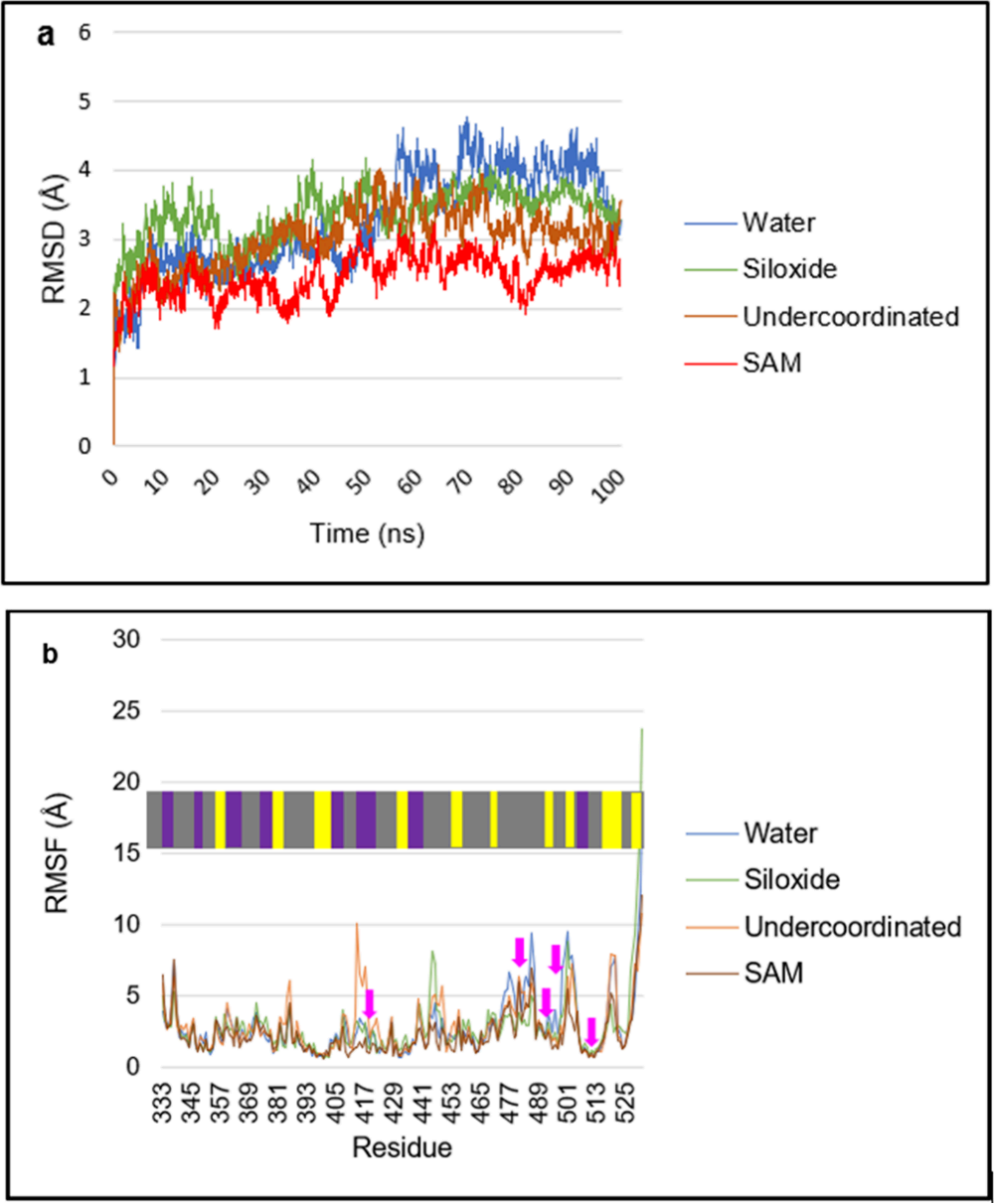
(a) RMSD and (b) RMSF of the His-Tag RBD for the various simulations in water. The colored ribbon at the top of the RMSF figure indicates the secondary structure of the protein: β-sheets (yellow) and α-helices (purple), while unstructured parts are shown in gray. The His-Tag is at residues 527–532, while the pink arrows indicate the location of the ACE2 binding residues.

Figure 4b reports the RMSF measured during the simulation. The RMSF values for the ACE2 binding residues are ∼3.1 Å for Lys417 (positive, hydrophilic), ∼3.2 Å for Asn487, ∼3.7 Å for Gln493, ∼9 Å for Gln498, and ∼9 Å for Tyr505. The RBD region of the S protein would be expected to have good conformational flexibility, as it has also been observed previously.^37^ The RMSF further shows that fluctuations are generally small at the secondary structure regions, and higher RMSF values generally occur at loop regions. The protein also maintains a higher RMSF at the His-Tag region, indicating that this has greater conformational flexibility and aligns with the visualization of the simulation trajectory that shows the His-Tag movement.

Visualization (see SI Movies) and overlap of the initial and final structure (Figure 3a) indicate the structure is well maintained with deviations observed mainly in the flexible loop regions, while the secondary structure elements, namely, α-helices and β-sheets, are well maintained. The spatial organization of α-helices and β-sheets also remains seemingly unchanged, as confirmed by the RMSF values in Figure 4b.

### 3.2 Negatively Charged Silica Surface

The overall positive charge of the protein means that adsorption on the negative silica siloxide surface is expected. In the early stages of simulation, the protein moved freely in the center of the simulation cell, with the electric field above the surfaces screened by the solution ions. The His-Tag, which was very flexible in its movements, extended in the fluctuating electric field to guide the protein to adsorb on the negative siloxide surface.

Having diffused closer to the surface, there followed rapid penetration of the water/ion surface layer by protein residues. The first anchoring event, where a residue’s side chain displaces the water layer closest to the silica surface, occurred at 44 ns by His 531 from the His-Tag, which is a positive and hydrophilic residue. His 531 was joined by Asn 370 (neutral, hydrophilic) and His 529 residues at the ion/water layer, with further adsorption of the Asn 37 at 48 ns and His 527 and His 530 residues at 50 ns. The protein seemed more stable in this simulation when compared with the water simulation (Figure 4a), maintaining a steady structure when adsorbed to the surface. This can be attributed to the positive His-Tag attraction to the negative surface, which anchors the protein rapidly. The adsorbing residues were followed closely by other nonadsorbing His-Tag residues, His 526–528, which did not interact directly with the surface.

Positively charged arginine and lysine residues, which might otherwise be expected to drive adsorption on negative surfaces, are concentrated in the center of the protein, and the protein has a hydrophilic core. The surrounding sides of the protein seem to be flexible with hydrophobic residues and an irregular distribution of charged residues, resulting in regions with varying partial charges. The positive ion layer shielding the surface also attracts negative residues, although adsorption seems to be driven by direct interaction of the positive His-Tag with the negative surface. In the final stable adsorption state, three His-Tag residues (His530, His531, His532) and Asn370 are adsorbed to the surface, as visualized in Figure 5. Despite its adsorption to the surface, the protein maintains an overall stable RMSF (Figure 4b), except in loop regions, where there is high fluctuation.

**Figure 5:**
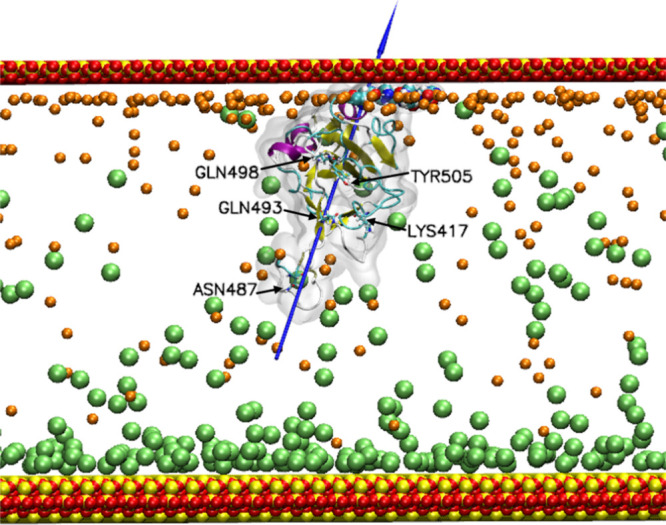
Adsorption of the His-Tag RBD on the siloxide-rich silica surface. The protein color scheme is the same as in Figure 2, and the outer layer of oxygen atoms in the silica model surface is shown as red spheres. Cl^–^ ions are shown as lime VdW spheres, and Na^+^ is shown as orange. The adsorbing residues are also shown in VdW representation, and the ACE2 adsorbing residues are indicated by the black arrows. The blue needle indicates the dipole moment, and water molecules are not shown for clarity.

The RMSF in Table 2 indicates that of the adsorbing residues, the His-Tag residues have higher conformational flexibility during the simulation prior to adsorption. The His-Tag continues to fluctuate on the surface once it adsorbs. The protein maintains an overall stable RMSD of ∼4 Å, as also seen in the water simulation (Figure 4a), and the structure overlaps (Figure 3b) confirm that the secondary structure remains intact after adsorption. During the course of this simulation, the protein’s dipole moment is directed toward the siloxide-rich surface, as expected with the positively charged His-Tag interacting with the negatively charged surface, and the final orientation is shown in Figure 5.

**Table 2:** RMSF of the Anchoring Residues on the Negatively Charged Surfaces

residue/simulation	RMSF (Å)
Asn 370 – siloxide	∼3
His 530 – siloxide	∼24
His 531 – siloxide	∼24
His 532 – siloxide	∼24
His 531 – SAM	∼12
His 532 – SAM	∼12

### 3.3 Model Positively Charged Silica Surface

The His-Tag protein is expected to be repelled by the positively charged surface. Consequently, it was initially positioned close to the model under-coordinated Si silica surface. The protein diffused freely and at 57 ns rotated to reverse its orientation; this event corresponds to an RMSD increase to ∼4 Å at 57 ns, followed by a reduction back to ∼3 Å at 76 ns (Figure 4a). Therefore, the protein did not adsorb to the positively charged surface model, and at the end of the 100 ns trajectory, the protein was diffusing in the center of the simulation cell. Consequently, its RMSD (Figure 4a) is in line with that for the protein in water, and indeed is similar in magnitude for the values found when the protein adsorbs to the negatively charged surfaces. Likewise, the RMSF values reported in Figure 4b are very similar across all cases. The structure overlaps (Figure 3c) confirm that the secondary structure of the protein remains intact throughout the simulation.

### 3.4 SAM Terminated Negatively Charged Surface

The protein, which has a net positive charge at pH 6, was placed in a side-on starting conformation with the His-Tag facing the lower negative surface. In the trajectory, the protein diffused and slowly moved toward the lower surface, and His532 residue adsorbed in the first anchoring event at 20 ns. The RMSD is slightly lower than that of the protein in water (Figure 4a), indicating good stability on adsorption. His531 residue also displayed a particular tendency to move downward and adsorbed to the surface at 28 ns. Interestingly, only these two of the six His-Tag residues adsorbed to the surface; the other four His-Tag residues did not adsorb in this trajectory. In fact, at 38 ns His532 desorbed, although it readsorbed at 47 ns.

The protein was stable (Figure 4a) in this adsorbed state over the 100 ns time scale. The ACE2 binding residues were also exposed to the bulk water in this simulation (Figure 6), implying that carboxyl-terminated SAMs provide a viable route for surface functionalization with the RDB.

**Figure 6:**
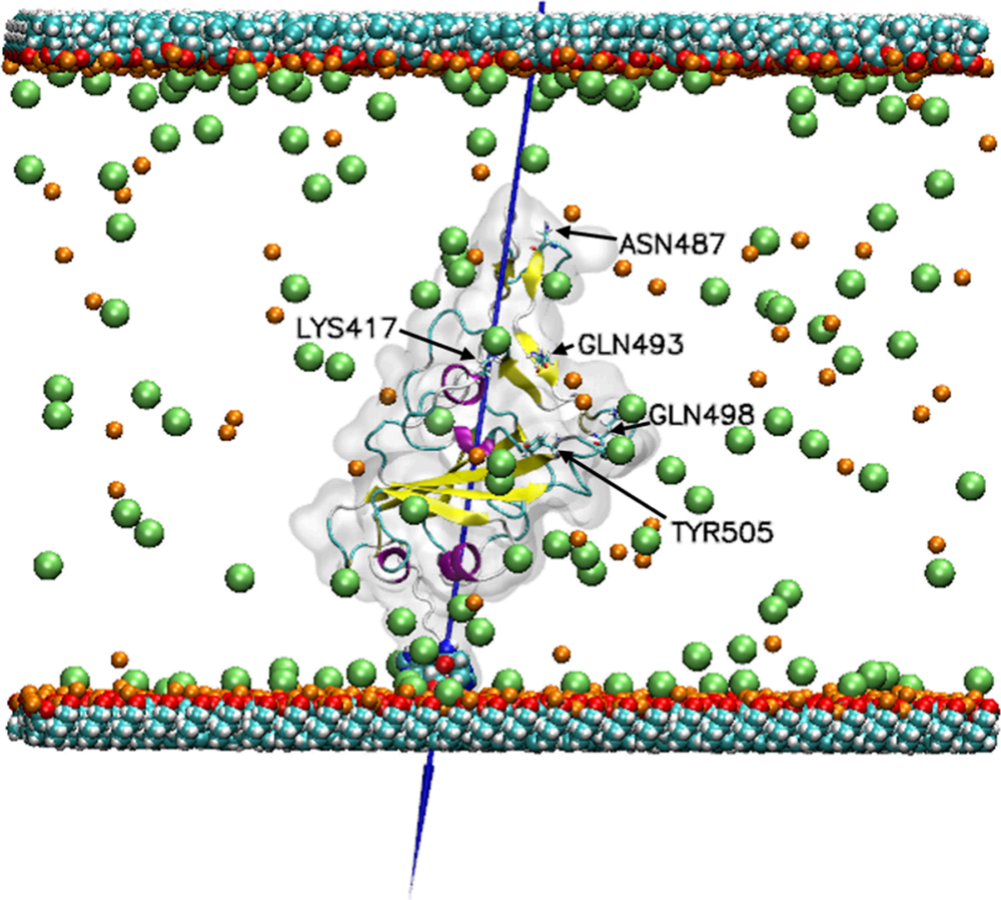
Adsorption of His-Tag RBD on a −COOH terminated SAM. The color scheme is the same as in Figure 5.

The anchoring residues in the SAM-terminated surface simulations were immobilized upon adsorption of the protein. The lack of rigidity of the substrate in the SAMs surface has a role to play, as, unlike the silica surface, where the atoms are fixed in space, the molecules that make up the SAMs-terminated surface are flexible.^38,39^ The SAM-terminated surface can therefore be considered as a ‘soft’ surface, which allowed penetration of the anchoring residues to create multiple interactions and thus inhibit further diffusion.^21^

The high charge density of the SAM surface attracts a strongly interacting layer of Na+ ions from solution to screen the electric field. The protein’s anchoring residue side chains had to first displace Na+ to enable adsorption. However, once this was done, it resulted in a small separation between the anchoring residues and charged functional groups on the SAM surface.

The RMSF values of the adsorbing residues, shown in Table 2, are lower for the SAM adsorption than for the siloxide-rich silica surface adsorption due to the greater reduction in side-chain flexibility following adsorption.

As is the case when adsorbed to the siloxide-rich silica surface, the protein maintains an overall stable RMSD (Figure 4a), while the structure overlap (Figure 3d) confirms that the secondary structure remains intact following adsorption to the SAM surface.

### 3.5 Stability of the His-Tag RBD Adsorbed on the Negatively Charged Surfaces

The adsorption simulations on negative surfaces were further assessed for protein stability and to establish their suitability in guiding the experimental work. The negative surface models were further analyzed as they are realistic and better representative of the protein binding to negative nanoparticles experimentally. Therefore, the adsorption simulations on the silica siloxide and COOH SAMs terminated surfaces were further assessed.

The number of intramolecular hydrogen bonds in the protein indicates its structural stability. Figure 7a shows that the number tends to be higher during the adsorption process but then settles back to the initial number, again reflecting the structural stability of the adsorbed protein. Specifically, the number is highest at ∼80 ns in the silica siloxide simulation when the His-tag adsorbs, and is highest at ∼20 ns in the SAM simulation when His532 adsorbs. The influence of a His-Tag on hydrogen bonding has also been observed previously.^40^

**Figure 7:**
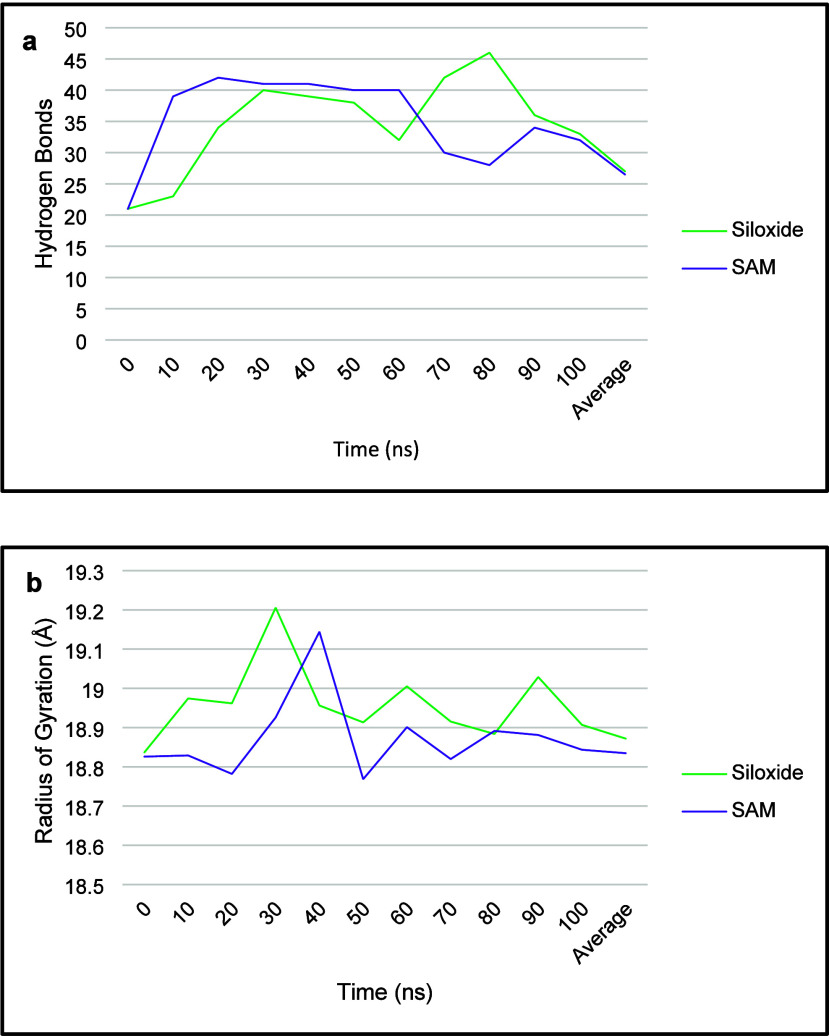
(a) Hydrogen bonds and (b) radius of gyration every 10 ns, and the average over 100 ns for the protein adsorption on silica siloxide and SAMs terminated surfaces.

The adsorbing residues His 531 and His 532 were the most active on the SAM surface, and as both of these residues are positive at pH6, and positive charges form stronger hydrogen bonds, this could result in more hydrogen bonds when compared with the silica siloxide simulation, which has one neutral and one positive active residue. Furthermore, the hydrogen bonding data for the adsorption simulations on negative surfaces agree with the adsorbed residues in the final state (Table 3). The role of a His-Tag in controlling protein surface orientation to position the protein as required has also been studied previously,^41^ and in this instance, it seems to act like a spacer arm to give the desired result.

**Table 3:** List of Adsorbed Residues in the Final Adsorption State of Adsorption Simulations of the Protein on Negative Surfaces

**simulation**	**residues**
silica siloxide surface	His 530, His 531, His 532, Asn 370
COOH SAM-terminated surface	His 531, His 532

The protein maintains a consistent RoG throughout the simulation in the silica siloxide simulation, with an average RoG of ∼18.9 Å (see Figure 7b). The data are also consistent with the RMSD data for this simulation (see Figure 4a), where the protein maintains an RMSD of ∼3.3 Å throughout. Likewise, the SAM surface simulation has an average RoG of ∼18.8 Å, indicating good stability of the protein in its adsorbed state as the simulation progresses. The RoG data is further complemented by the RMSD data (see Figure 4a), which shows the protein maintains an RMSD of ∼2.3 Å during the course of simulation, indicating good overall structural integrity.

## 4 Conclusions

In this study, fully atomistic MD simulations of the RBD of the SARS-CoV-2 S protein, modified with a His-Tag, at different model surfaces are presented. The simulation of the protein with the negative silica siloxide surface showed anchoring was rapid and specific, while resulting in a ‘head-on’ final conformation. The protein simulation with the positive silica undercoordinated surface showed just free diffusion in the center of the system for the duration of the simulation. However, the protein moved rapidly to the negative SAM-terminated silica surface and was quick to adsorb. The His-Tag played a crucial role in driving the RBD toward the negative surfaces, especially on the SAM surface, acting like a spacer arm to drive adsorption. Consequently, the His-Tag residues play a dominant role in all simulations and play an active role in the adsorption of the protein to both negative surfaces. The RMSD data indicated the protein maintained a good level of stability in all simulations, and the RMSF was generally small in all simulations. This suggests overall that the secondary structure was well-maintained in all simulations, as evidenced by the trajectories and further supported by visual overlaps of the structures. The ACE2 binding residues were largely unconstrained in both adsorption simulations on the experimentally relevant negative model silica siloxide and model SAMs surfaces, opening up the possibility of the development of new therapeutics.

The aforementioned results indicate that adsorption to the negatively charged silica surface, as observed experimentally for silica nanoparticles, is likely to produce favorable S protein RBD adsorption that facilitates the binding of the ACE2 receptor. However, it is important to recognize that these fully atomistic simulations only display the initial stages of protein adsorption, rather than the long-term protein configuration relevant to the experiment. Nevertheless, the proteins show good stability once adsorbed, at least for the time scales used here, as seen in the accompanying movies. Therefore, we believe these simulations show encouraging pathways for the design and delivery of new therapeutics not only for COVID-19, but also for other viral threats.

## Supplementary Material



## Notes

100 ns trajectories for protein simulation in water, negative silica surface, positive silica surface, and negative SAM-terminated surface (ZIP)
